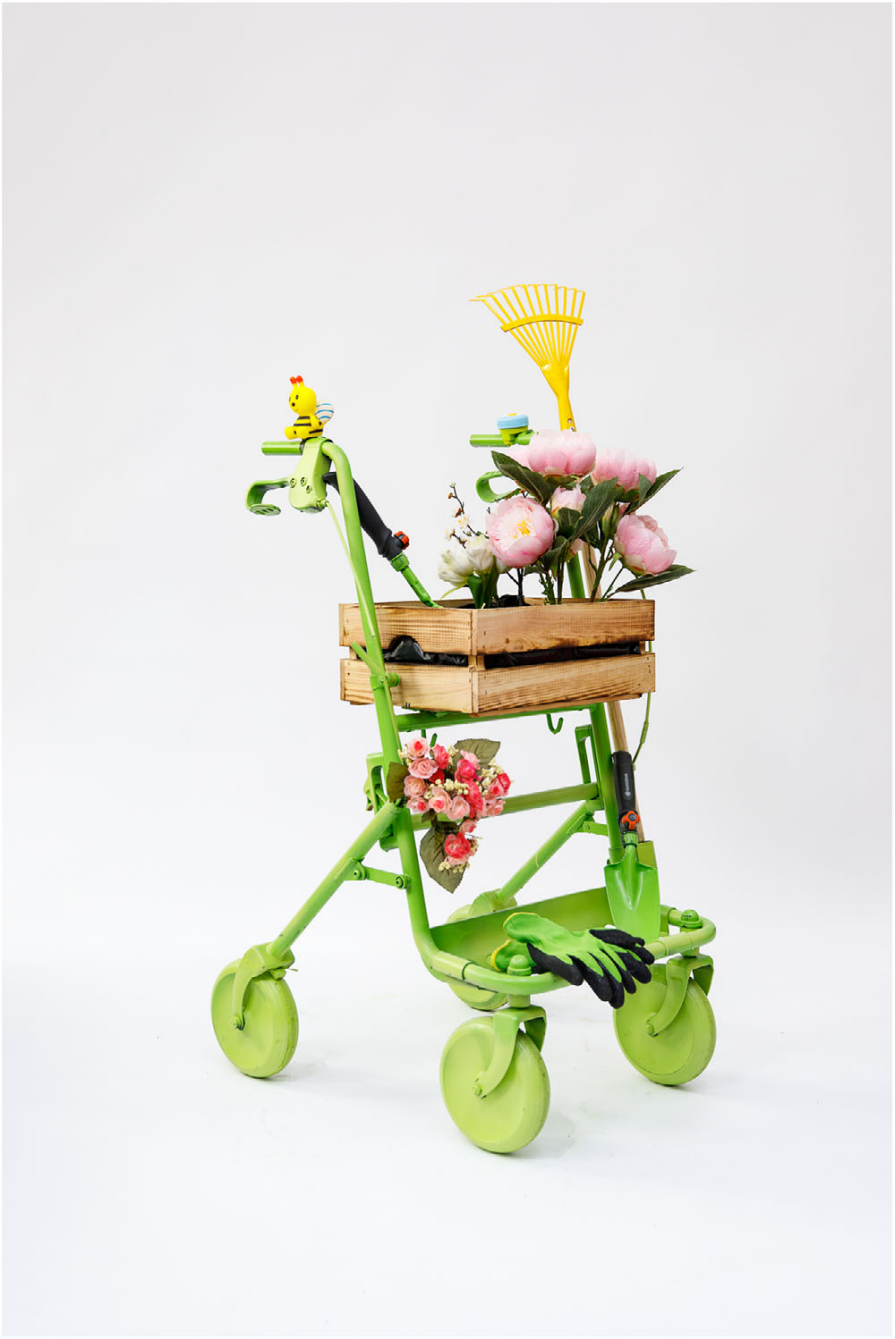# Demedarts

**DOI:** 10.1002/alz.084686

**Published:** 2025-01-09

**Authors:** Ruth Gabriele Mateus‐Berr, Pia Moana Scharler‐Plotnik, Lisa Kielmeier, Alexandra Rusz

**Affiliations:** ^1^ University of Applied Arts Vienna, Vienna, Vienna Austria; ^2^ University of Applied Arts Vienna, Vienna, Vianna Austria

## Abstract

This rollator/walker called THE GARDENER is a so‐called Critical Design. it is not intended for actual use but to attract attention, to encourage new interpretations for designs for those affected. in spring and summer you can go for a walk with a raised bed, so to speak, and talk to/interact with other passers‐by, thus overcoming the loneliness of old age. Hapitically and olfactorily you have a positive connotation.